# Preparation, Characterization and In Vitro Evaluation of Eudragit S100-Coated Bile Salt-Containing Liposomes for Oral Colonic Delivery of Budesonide

**DOI:** 10.3390/polym14132693

**Published:** 2022-06-30

**Authors:** Hamid Alghurabi, Tatsuaki Tagami, Koki Ogawa, Tetsuya Ozeki

**Affiliations:** 1 Drug Delivery and Nano Pharmaceutics, Graduate School of Pharmaceutical Sciences, Nagoya City University, 3-1 Tanabe-dori, Mizuho-ku, Nagoya 467-8603, Japan; hamid.alghurabi@gmail.com (H.A.); tagami@phar.nagoya-cu.ac.jp (T.T.); kogawa@phar.nagoya-cu.ac.jp (K.O.); 2 Department of Pharmaceutics, College of Pharmacy, University of Kerbala, Kerbala 56001, Iraq

**Keywords:** polymer-coated liposomes, sodium glycocholate, colonic-targeted delivery, pH sensitive polymer

## Abstract

The aim of this study was to prepare a liposomal formulation of a model drug (budesonide) for colonic delivery by incorporating a bile salt (sodium glycocholate, SGC) into liposomes followed by coating with a pH-responsive polymer (Eudragit S100, ES100). The role of the SGC is to protect the liposome from the emulsifying effect of physiological bile salts, while that of ES100 is to protect the liposomes from regions of high acidity and enzymatic activity in the stomach and small intestine. Vesicles containing SGC were prepared by two preparation methods (sonication and extrusion), and then coated by ES100 (ES100-SGC-Lip). ES100-SGC-Lip showed a high entrapment efficiency (>90%) and a narrow size distribution (particle size = 275 nm, polydispersity index < 0.130). The characteristics of liposomes were highly influenced by the concentration of incorporated SGC. The lipid/polymer weight ratio, liposome charge, liposome addition, and mixing rate were critical factors for efficient and uniform coating. In vitro drug release studies in various simulated fluids indicate a pH-dependent dissolution of the coating layer, and the disintegration process of ES100-SGC-Lip was evaluated. In conclusion, the bile salt-containing ES100-coated liposomal formulation has potential for effective oral colonic drug delivery.

## 1. Introduction

Colon-targeted oral drug delivery has received growing attention over the past few decades for the treatment of both local and systemic conditions. This region of the gastrointestinal tract (GIT) has several unique properties that make it advantageous compared to the stomach and small intestine in terms of formulation stability, release, and permeation (e.g., near neutral pH, low enzymatic activity, low bile salt concentrations, long residence time, and slow secretion of mucus) [1]. These properties have established the colon as a potential target for the systemic delivery of degradable and poorly permeable biopharmaceuticals such as protein and peptide drugs via the oral route, which is widely accepted as the most efficient, convenient, and cost-effective route for drug administration [2,3]. For local treatment of colonic pathological conditions (e.g., inflammatory bowel syndrome, ulcerative colitis, colorectal cancer), controlling the site of drug release throughout the GIT provides higher drug bioavailability at the target site, which in turn increases therapeutic efficacy and reduces the administered dose and systemic side effects [4].

Liposomes have been extensively researched as drug delivery carriers for many years since they can provide control of the rate and/or site of release for a wide range of drugs with different physiochemical properties. Currently, the main clinical applications for liposomes are the treatment of severe systemic infections and cancer [5]. However, for these applications, it is usually necessary to deliver the drug via the parenteral route [6]. There are several studies demonstrating the ability of liposomes to interact favorably with healthy and inflamed colonic mucosa in vitro, which further proves their value in colonic drug delivery [7,8,9,10]. However, for colonic action, the administration of liposomes in all these studies was either intraluminal or in vitro to excised tissue; delivery via oral administration was not attempted. Colonic drug delivery through the oral route has several obstacles, of which the passage of the dose through regions of high acidity and digestive activity is the main one. Conventional liposomes by nature are not suitable for oral drug delivery since they are susceptible to digestion in various regions of the GIT by gastric acid, pancreatic lipase, and intestinal bile salts [11]. Therefore, a suitable formulation for delivering liposomes orally can extend their clinical applications and open new possibilities for therapy.

Various strategies have been utilized to improve the stability of liposomes in the harsh GIT environment, such as coating liposomes with polymers (e.g., chitosan and polyethylene glycol) [12,13,14], but few studies have specifically targeted the colonic region. Several studies have aimed for oral delivery of liposomes to the colon by coating them with the methacrylate copolymer Eudragit S100 (ES100) [15,16,17]. The ES100 coating allowed pH-dependent drug release to be achieved; however, the formulations in these studies were either not tested against physiological bile salts [17], not able to protect liposomes from bile salt [15], or not economically viable [16].

Bile salts, physiological surfactants secreted by hepatocytes, are the main cause of the destruction of liposomes in the GIT, as they form colloidal mixed micelles with the phospholipids of lipid bilayers [18,19,20,21]. Surprisingly, studies have shown that prior incorporation of bile salts such as sodium glycocholate (SGC), sodium taurocholate (STC), and sodium deoxycholate (SDC) into the liposome bilayer structure stabilizes the membranes against the damaging effect of physiological bile salts [19,22,23,24,25,26]. Liposomes and niosomes containing bile salts have been widely researched for both oral immunization [23,27] and oral delivery of biomacromolecules and drugs with poor water solubility [25,26,28]. The stability gained by the inclusion of bile salts into vesicles has been attributed to the repulsion between the bile salts that preexist in the vesicle bilayer and intestinal bile salts in the GIT [19].

**Figure 1:**
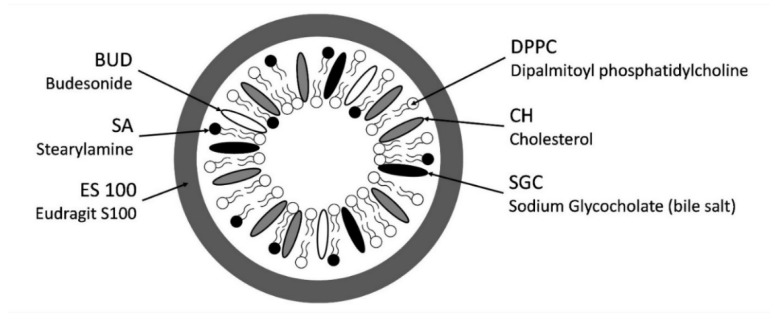
Schematic diagram for ES100-coated bile salt-containing liposomes.

In the present study, our goal was to prepare a colon-targeted drug delivery system for an anti-inflammatory model drug, budesonide (BUD), based on liposomes that are resistant to physiological bile salt. The drug delivery system involves a combination of incorporating bile salts into the liposomal membrane and coating the liposomal surface with a pH-sensitive polymer (Figure 1).

To the best of our knowledge, this is the first liposomal formulation that combines both bile salt integration and ES100 coating for oral colonic drug delivery. We selected the methacrylate copolymer ES100 as the coating material due to its previous successful use in the development of a commercially available tablet for colonic drug delivery [29] and its promising potential for delivering liposomes to the colon [16,17,30]. This anionic polymer has a solubility threshold of pH 7, which renders it insoluble at lower pH values in the stomach and upper small intestine while allowing drug release from coated liposomes at the junction between the small intestine and colon where a pH level of 7 occurs. This can achieve targeted drug release in the distal small intestine and the colon where digestive enzyme and bile salt concentrations are low [31]. Stearylamine (SA) was incorporated into the structure of the liposomes to create a positive surface charge that facilitates coating the liposomes with ES100 due to the electrostatic attraction between the cationic liposomes and the anionic polymer [32]. To overcome the emulsifying effect of physiological bile salts in the small intestine on the structural integrity of liposomes, SGC (a bile salt) was added to the lipid during liposome formulation.

## 2. Materials and Methods

### 2.1. Materials

BUD, dipalmitoylphosphatidylcholine (DPPC), SA, SGC, and STC were purchased from Tokyo Chemical Industry (Tokyo, Japan). Cholesterol (CH) was obtained from Wako Pure Chemical Industries, Ltd. (Osaka, Japan). ES100 was donated from Evonik (Essen, Germany).

### 2.2. Methods

#### 2.2.1. Formulation of Liposomes

Liposomes were prepared using DPPC, CH, and SA. Multilamellar vesicles were produced by the thin film hydration method, from which large unilamellar vesicles were produced as described below. Briefly, the lipids (20 mM) were dissolved along with 0.25 mg of BUD into 1 mL of chloroform in DPPC:CH:SA molar ratios of 7:3:X, 8:2:3, or 6:4:3 (where X represents various molar ratios of SA: 0, 0.25, 0.5, 1, 2, 3, and 4) as shown in Table 1. The organic solvent was removed under a light stream of nitrogen at 65 °C. The lipid film was further dried by storing the flask overnight in a vacuum desiccator. The dried film was then hydrated with 1 mL of different SGC concentrations [24,27] (0, 0.25, 0.5, and 1 mg/mL) in 5% dextrose solution at 65 °C and swirled for 10 min to form a dispersion.

Homogenization of liposomes was performed either by ultrasonication or extrusion. Ultrasonication was performed by a Bioruptor^®^ UCD-250 (Cosmo Bio, Tokyo, Japan) in which the particle size was reduced by 15 cycles of ultrasonication (50 s on/10 s off) at 250 W while maintaining the temperature at 4 °C by using an ice bath. The tube holder was always filled completely with 1.5 mL Eppendorf tubes to ensure the homogeneity of sonication, and sample volumes of 500 µL were used in each tube to maximize sonication efficiency [33].

Extrusion was performed using an Avanti Mini-Extruder (Avanti Polar Lipids, Inc.; Alabaster, AL, USA), via which the liposomes were passed through 200-nm filter polycarbonate membranes (Whatman^®^ Nuclepore membrane; Whatman Inc., Florham Park, NJ, USA) 20 times at a temperature of 65 °C to create vesicles with a narrow size distribution. Free unentrapped precipitated drug was removed by using gentle centrifugation (2000× *g*, 5 min) [34].

#### 2.2.2. Coating of Liposomes

Liposomes were coated with ES100 using the method described by Henriksen [35,36]. Several coating variables were investigated to optimize the coating process: liposomal concentration, ES100 concentration, liposome/polymer volume ratio, rate of liposomes addition, and stirring speed. Liposomes at different concentrations (2, 4, 5, 7, 10 mM) were combined with aqueous solutions of ES100 at different concentrations (0.25, 0.5, 1, 2, 2.5 mg/mL in 100 mM phosphate buffer, pH 8.0) in different liposome/ES100 volume ratios (1:1, 1:2, 1:3). The liposomes were added dropwise at different rates (5, 10, 50, 250 µL/s) to the ES100 solution placed in an ice bath while stirring at different speeds (100, 500, 1000 RPM) at 4 °C by using an ice bath for 30 min. The resulting mixture was centrifuged through a membrane filter (Amicon Ultra-2 MWCO = 100 kDa, Millipore, Ireland) at 7500× *g* for 10 min at 4 °C, after which the coated liposomes were then resuspended in 5% dextrose (pH 6).

#### 2.2.3. Entrapment Efficiency (EE) Determination

To determine the EE, purified BUD liposomes after centrifugation (2000× *g*, 5 min) were dissolved in methanol in a 1:10 volume ratio to release BUD from the lipid. The content of the drug within the liposomes (Drug_Entrapped_) was analyzed by UV spectrophotometry at 243 nm as described in the UV assay section. Total drug was determined by dissolving the liposome sample in methanol before centrifugation. The EE of the drug in the liposomes was determined by the Equation (1)

Entrapment Efficiency % = (Drug_Entrapped_/Drug_Total_) × 100% (1)

#### 2.2.4. Particle Size Measurements

The vesicle size distribution represented by the Z-average size (Z-avg) and the polydispersity index (PDI) of different liposomal preparations was obtained from dynamic light scattering (DLS) measurements using a Zetasizer Nano-S ZEN1600 (Malvern Instruments Limited, Malvern, UK). Before each analysis, samples were diluted in distilled water (viscosity of 0.8872 cP, refractive index of 1.330). The tested samples (0.8 mL) were measured in disposable polycarbonate cuvettes at temperature of 25 °C by recording the scattered light signal at a fixed angle of 90°. The Z-avg was calculated by Zetasizer software version 7.13 (Malvern Panalytical; Malvern, UK) based on the fluctuation in the intensity of the scattered light caused by the Brownian movement of particles with different sizes. The obtained results were the average of three measurements.

#### 2.2.5. Zeta Potential Measurements

Zeta potential measurements were carried out with a Zetasizer Nano ZS90 (Malvern Instruments Limited). Samples were diluted with distilled water, then measured at 25 °C in a 1.0 mL polycarbonate cuvette, DTS1070. Measurements were taken for three independent samples of each formulation.

#### 2.2.6. Drug Release Study

In vitro drug release of liposomal formulations was tested in different release media, each simulating a different section of the GIT: simulated gastric fluid (SGF) represents the stomach, fasted-state and fed-state simulated intestinal fluids (FaSSIF and FeSSIF, respectively) represent the small intestine, and phosphate buffer saline (PBS) pH 7.4 represents the colonic region. The composition of each release medium is shown in Table 2.

Different liposomal suspensions (1 mL, equivalent to 20 μg/mL of BUD, below saturated solubility) were added to 9 mL of each release medium preheated at 37 °C and agitated thoroughly in a water bath shaker with horizontal shaking (approximately 100 rpm) maintained at 37 °C. Samples of 1 mL were withdrawn at predetermined time points (0.5, 1, 2, 4, 8, 12, and 24 h) and then ultracentrifuged (300,000× *g* for 20 min) to pellet the liposomes. The supernatant was analyzed by UV spectrophotometry against a standard calibration curve obtained at 247 nm to determine the concentration of the released drug as described in the UV assay section. Each measurement was taken against a sample of blank liposomes of the corresponding liposomal formulation in the relevant release medium. Before release, the initial drug concentration in each formulation was determined by lysing the liposomes with methanol and analyzing the drug concentration using UV spectroscopy, then drug release was calculated as a percentage of the total drug entrapped.

Changes in liposomes particle size for each formulation was also measured during incubation at different release media. Each formulation was incubated in release media (SGF, FaSSIF, FeSSIF, or PBS pH = 7.4) at 37 °C in a shaking water bath. At the predetermined time points (0, 0.5, 1, 2, 4, 8 h), samples were collected, and the particle size was measured. TEM and FTIR examination were also conducted to monitor changes in the morphology and structure of the liposomes after 2 h of incubation in each release medium.

#### 2.2.7. Stability Study

The stability of BUD-loaded liposomes was tested by incubating the liposomal formulation at two different temperatures: 4 °C and 25 °C. Samples were withdrawn at predetermined time intervals (0, 24, 48, 72 h for 25 °C samples, and 0, 1, 2, 3, 4, 5 weeks for 4 °C samples) to measure particle size by DLS and drug content concentration by UV. Each experiment was performed in triplicate.

#### 2.2.8. Transmission Electronic Microscopy (TEM)

The uncoated/coated liposome shape and surface morphology was examined by TEM. Samples were prepared by placing a 200-mesh copper grid coated with carbon on a drop of the liposome preparation and waiting for 5 min to allow for sample deposition on the grid. Negatively stained TEM samples were prepared by dipping the liposomes-loaded grid on a 2% (*w/v*) uranyl acetate solution for another 5 min. Excess sample and reagent were removed from the grid using a filter paper, then the grids were left to dry completely. Micrographs were recorded using a Jeol JEM-1400 Plus microscope (JEOL Ltd.; Tokyo, Japan) working at an accelerating voltage of 100 kV.

#### 2.2.9. UV Assay

BUD concentration was determined by UV spectrophotometry (UV-1800, Shimadzu, Tokyo, Japan) against a standard curve using serial concentrations of BUD in each release medium (2–10 µg/mL, R^2^ > 0.99) for the aqueous solutions obtained at 247 nm, and (0–50 µg/mL, R^2^ > 0.99) for the methanolic solutions obtained at 243 nm. For the aqueous solutions, a stock solution of 1000 mg/mL BUD in methanol was first prepared and then diluted in the corresponding release media to obtain a 10 µg/mL aqueous stock solution. All measurements were taken against blank samples of the corresponding release media.

#### 2.2.10. Fourier Transform Infrared Spectroscopy (FTIR)

The IR spectra of the freeze-dried liposomal formulations were obtained using an FTIR spectrophotometer (FTI-8400S, Shimadzu, Tokyo, Japan) as below. In brief, the optimized liposomal dispersions were frozen in 5% dextrose (also acts as cryoprotectant) at −80 °C for 24 h in an ultra-low temperature freezer. Liposomal dispersions incubated in simulated media were ultracentrifuged (100,000× *g* for 60 min) and washed with 5% dextrose before freezing. The frozen liposomes were freeze-dried (FD-1000, EYELA, Tohoku, Japan) for 48 h.

Then, freeze-dried liposomes (2 mg) were blended with potassium bromide (spectroscopic grade) (100 mg), then compressed into disks by a hydraulic press before being scanned from 4000 to 500 cm^−1^ at a resolution of 4 cm^−1^. Data were analyzed using FTIR software (IRsolution version 1.10, Shimadzu, Tokyo, Japan).

#### 2.2.11. Statistical Analysis

Statistical analyses were performed using Prism Version 9 (GraphPad Software, San Diego, CA, USA). All results are expressed as means ± standard deviation. Statistical analyses were performed using t-test and one-way or two-way ANOVA (depending on the number of independent variables) followed by Tukey’s test to evaluate significant differences between different groups. Values of *p* < 0.05 were considered statistically significant.

## 3. Results

### 3.1. Effect of SA on Liposome Particle Size, PDI, EE, and Zeta Potential

**Figure 2:**
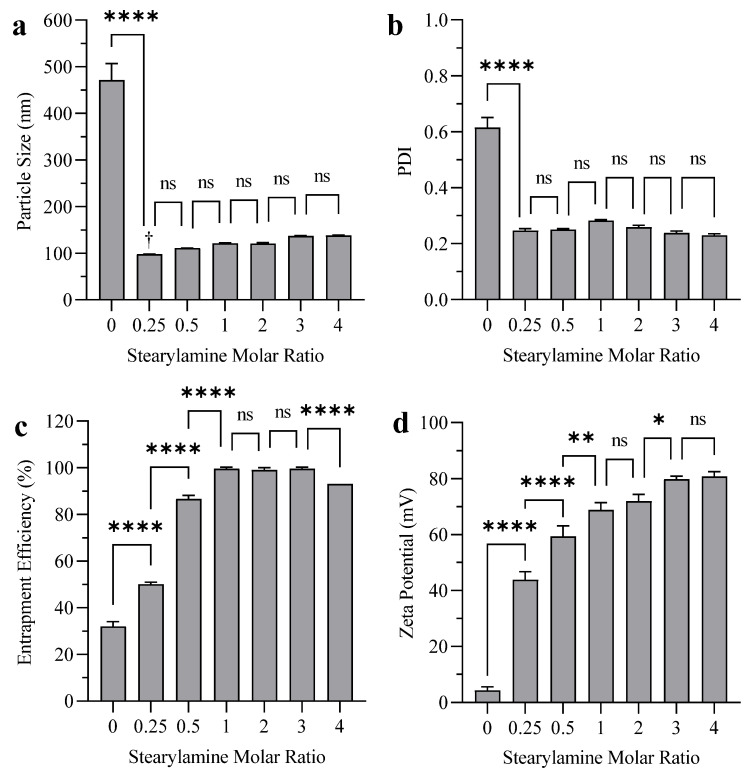
The effect of SA molar ratio on the (**a**) particle size (**b**) PDI (**c**) EE, and (**d**) zeta potential of budesonide liposomes with DPPC:CH:SA molar ratio of 7:3:X (where X represents various molar ratios of SA: 0, 0.25. 0.5, 1, 2, 3 and 4) without bile acid prepared by sonication. **Notes:** The data represent the mean ± standard deviation (n = 3). (ns *p* ≥ 0.05, * *p* < 0.05, ** *p* < 0.01, **** *p* < 0.0001) compared to adjacent formulation(s). †: Small degree of aggregation was detected visually and by DLS.

Figure 2a,b shows the effect of SA inclusion into liposomes on the particle size and PDI. Liposomes without SA showed extensive aggregation, while liposomes with 0.25 (2.4 mol%) molar ratio of SA showed a smaller degree of aggregation with much lower particle size and PDI. Increasing the molar ratio of SA to 0.5 (5 mol%) and above prevented the aggregation of liposomes; however, there was no significant effect on the particle size and PDI. The results showed an increase in BUD EE as the SA molar ratio increased (Figure 2c) from 0 to 1 (9 mol%). Increasing the SA molar ratio further had no effect on EE until the SA molar ratio was above 3 (23 mol%), where the EE of the drug decreased again. Zeta potential increased significantly from +4 mV to +80 mV when the SA molar ratio was increased from 0 to 3 (23 mol%). Higher levels of SA were not found to significantly increase zeta potential (Figure 2d).

### 3.2. Effect of Bile Salt and CH Content on Liposome Particle Size, EE, and Zeta Potential

**Figure 3:**
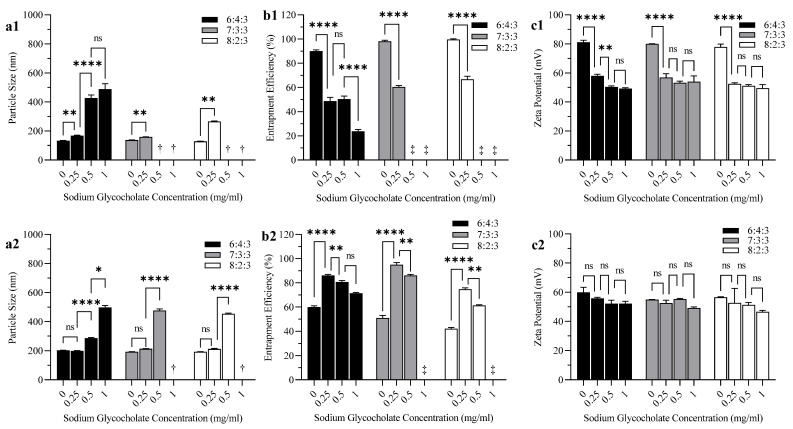
The effect of SGC (bile salt) concentration (mg/mL) on the (**a1**,**a2**) particle size, (**b1**,**b2**) EE, and (**c1**,**c2**) zeta potential of liposomes with DPPC:CH:SA molar ratios of 6:4:3, 7:3:3, and 8:2:3 prepared by sonication (**a1**–**c1**) and extrusion (**a2**–**c2**). **Notes:** The data represents the mean ± standard deviation (n = 3). (ns *p* ≥ 0.05, * *p* < 0.05, ** *p* < 0.01, **** *p* < 0.0001) compared to adjacent formulation(s). †: Results do not meet DLS criteria (not included in two-way ANOVA). ‡: Precipitated drug cannot be separated from liposomes due to the large particle size of the liposomal vesicles (>1000 nm).

Figure 3 shows the effects of SGC on the properties of three liposomal formulations with various DPPC:CH molar ratios (6:4, 7:3, and 8:2) prepared by sonication and extrusion. Increasing the SGC concentration increased the particle size of liposomes significantly in all three formulations that were prepared by sonication and those prepared by extrusion (Figure 3(a1,a2)). The effect of the CH molar ratio on particle size was not significant; however, when SGC concentration was increased, liposomal formulations with DPPC:CH molar ratios of 7:3 and 8:2 started to display extensive aggregation compared to the 6:4 DPPC:CH molar ratio formulation. This effect was more noticeable in formulations prepared by sonication (Figure 3(a1)) compared to others prepared by extrusion (Figure 3(a2)).

All liposomal formulations prepared by sonication showed high EE (≥90%) before the addition of bile salt (Figure 3(b1)), while formulations prepared by extrusion showed much lower EE (≤60%) (Figure 3(b2)). In liposomes prepared by sonication, ANOVA results showed that increasing the SGC concentration decreased the EE of BUD significantly (*p* < 0.0001) in all three liposomal formulations (Figure 3(b1)). In contrast, the inclusion of SGC in liposomes prepared by extrusion increased the EE of BUD significantly (*p* < 0.0001) in all liposomal formulations. The effect of CH on EE was similar to that of SGC. Formulations with the lowest CH molar ratio (8:2:3) prepared by sonication showed the highest EE (Figure 3(b1)), while the same formulations prepared by extrusion showed the lowest EE (Figure 3(b2)).

The effect of SGC concentration on the zeta potential of liposomes was dependent on the method of preparation (sonication or extrusion). In formulations prepared by sonication, the incorporation of SGC decreased the zeta potential significantly (*p* < 0.0001) in all three formulations (Figure 3(c1)), while in formulations prepared by extrusion, there was no significant change in zeta potential when SGC was incorporated (Figure 3(c2)). Zeta potential measurements did not show any significant change in response to varying CH molar ratios in liposomal formulations regardless of the method of homogenization (sonication or extrusion).

### 3.3. Effect of Drug/Lipid Weight Ratio on Liposome EE

Figure S1 shows that the EE of BUD increased from 29% to 95% upon decreasing the drug/lipid weight ratio from 1:10 to 1:40. In other words, the higher the lipid ratio, the higher the EE.

### 3.4. Liposome Coating

**Figure 4:**
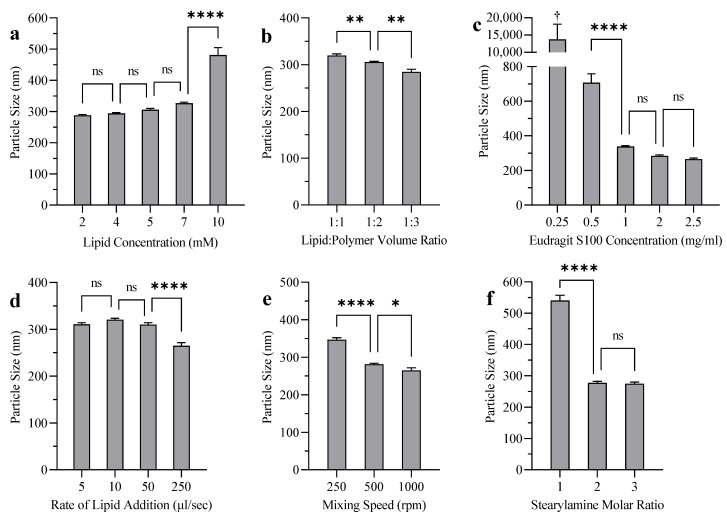
The effect of various coating variables: (**a**) lipid concentration, (**b**) lipid–polymer volume ratio, (**c**) ES100 concentration, (**d**) rate of lipid addition, (**e**) mixing speed, (**f**) SA molar ratio on the particle size of liposomes. **Notes:** The data represents the mean ± standard deviation (n = 3). (ns *p* ≥ 0.05, * *p* < 0.05, ** *p* < 0.005, **** *p* < 0.0001) compared to adjacent formulation(s). †: Results do not meet DLS criteria (not included in one-way ANOVA).

Figure 4, Figures S2 and S3 show the effects of coating parameters on the particle size, PDI, and zeta potential of liposomes after coating, respectively. Increasing the initial lipid concentration before coating increased particle size (Figure 4a) and PDI (Figure S2a) after coating significantly as a result of liposomal aggregation. The coated liposomes maintained their uniformity at lipid concentrations below 7 mM, and aggregation was visible at lipid concentrations around 10 mM. Decreasing the liposome/ES100 ratio decreased particle size significantly (Figure 4b), with no effect on the PDI (Figure S2b). Increasing the ES100 concentration decreased particle size (Figure 4c) and PDI (Figure S2c) significantly. Aggregation was visible at an ES100 concentration of 0.25 mg/mL. Adding liposomes faster to the coating solution decreased particle size (Figure 4d) and PDI (Figure S2d) significantly. There was no significant difference in particle size and PDI when the rate of addition was slowed to 50 µL/s and below. Increasing the stirring rate was associated with a decrease in particle size (Figure 4e) and PDI (Figure S2e). Finally, the incorporation of more SA content into the liposomes decreased particle size (Figure 4f) and PDI (Figure S2f) significantly. The coating caused a surface charge reversal in which the zeta potential was decreased from +53 mV to −38 mV (Figure S3). Zeta potential measurements did not show a significant difference upon changing the coating variables, except for the polymer concentration (Figure S3c). There was a slight decrease (*p* < 0.05) in EE after coating from 95% ± 2% to 91% ± 1%.

Figures S4 and S5 show FTIR spectra and TEM images of liposomes before the incorporation of bile salt (Lip), after the incorporation of bile salt (SGC-Lip), and after coating with the ES100 layer (ES100-SGC-Lip).

### 3.5. Release Data

**Figure 5:**
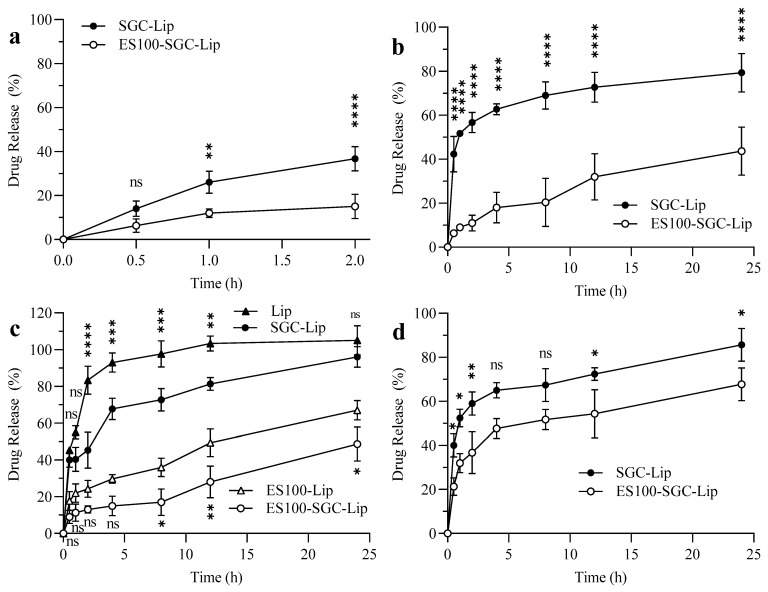
In vitro drug release of different liposomal formulations: SGC-Lip and ES100-SGC-Lip in (**a**) SGF (pH = 1.2), (**b**) FaSSIF (pH = 6.5), (**c**) FeSSIF (pH = 5), and (**d**) PBS (pH = 7.4). **Notes:** The data represent the mean ± standard deviation (n = 3). Two-way ANOVA followed by Tukey test were used for statistical analysis and comparison. In (**c**), two additional formulations, Lip and ES100-Lip, were also tested in FeSSIF. In (**a**,**b**,**d**), Tukey test was conducted to compare SGC-Lip with ES100-SGC-Lip formulations, except for (**c**) where Tukey test was conducted to compare Lip with SGC-Lip and ES100-Lip with ES100-SGC-Lip formulation. n = 3. (ns *p* ≥ 0.05, * *p* < 0.05, ** *p* < 0.005, *** *p* < 0.001, **** *p* < 0.0001).

The drug releases of BUD from SGC-Lip and ES100-SGC-Lip formulations in all four release media, SGF, FaSSIF, FeSSIF, and PBS pH 7.4, are summarized in Figure 5. Around 35% and 60% of the drug was released from SGC-Lip after a 2-h incubation in SGF (Figure 5a) and 4-h incubation in FaSSIF (Figure 5b), respectively. Regarding ES100-SGC-Lip, only 15% and 20% of the drug was released within 2 h in SGF and 4 h in FaSSIF, respectively. In FeSSIF (Figure 5c), Lip and SGC-Lip appeared visually to be completely destroyed after 1 h of drug release. However, there was a significant difference in the amount of drug released between the two formulations, where SGC-Lip retained the drug longer than Lip, before complete release. Both ES100-coated formulations with and without bile salt (ES100-SGC-Lip and ES100-Lip, respectively) retained their liposomal structure and size distribution (no signs of aggregation, precipitation, or change in opacity) in FeSSIF, but they showed similar differences in the extent of drug release observed between SGC-Lip and Lip. ES100-SGC-Lip showed only 15% drug release compared to ES100-Lip, which showed about 30% release after a 4-h incubation in FeSSIF. At pH 7.4, SGC-Lip and ES100-SGC-Lip showed similar release profiles, where about 65–85% was released within 24 h (Figure 5d).

### 3.6. Liposomal Integrity

**Figure 6:**
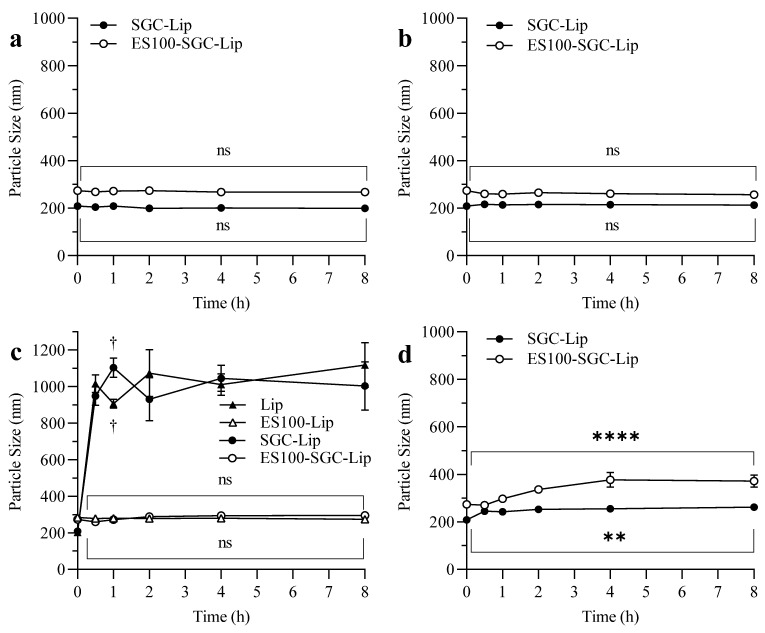
Particle size changes of different liposomal formulations: SGC-Lip and ES100-SGC-Lip in (**a**) SGF (pH = 1.2), (**b**) FaSSIF (pH = 6.5), (**c**) FeSSIF (pH = 5), and (**d**) PBS (pH = 7.4). Notes: In Figure (**c**), two additional formulations, Lip and ES100-Lip, were also tested in FeSSIF. Tukey test was conducted to compare the particle size of the liposomal formulations before and after incubation for 8 h in the release media. n = 3. (ns *p* ≥ 0.05, ** *p* < 0.005, **** *p* < 0.0001). †: Results do not meet DLS criteria due to liposomal destruction by bile salt.

Particle size measurements after incubation in different release media are illustrated in Figure 6. There was no change in the particle size of SGC-Lip and ES100-SGC-Lip in SGF and FaSSIF (Figure 6a,b). In FeSSIF (Figure 6c), only ES100-coated formulations (ES100-Lip and ES100-SGC-Lip) maintained their particle size, while uncoated formulations (Lip and SGC-Lip) lost their liposomal structure. In PBS pH 7.4 (Figure 6d), SGC-Lip particle size increased rapidly from 209 to 262 nm within the first 30 min, while ES100-SGC-Lip particle size increased gradually from 274 to 372 nm within 60 min.

**Figure 7:**
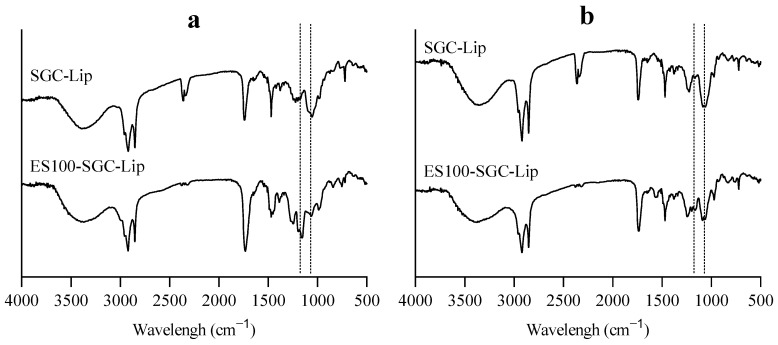

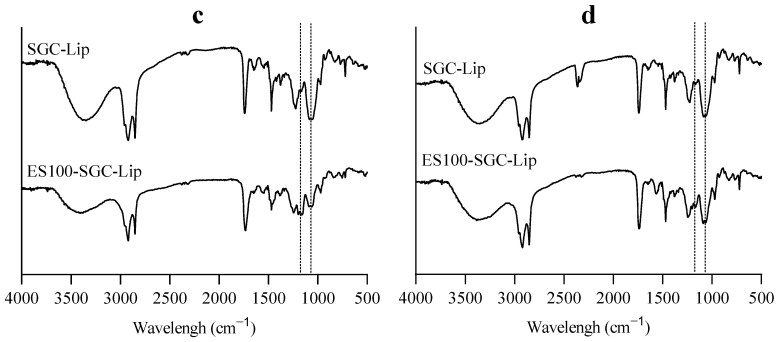
FTIR spectra of SGC-Lip and ES100-SGC-Lip obtained after 2-h incubation at 37 °C in (**a**) SGC pH 1.2, (**b**) FaSSIF pH 6.5, (**c**) FeSSIF pH 5, (**d**) PBS pH 7.4.

**Figure 8:**
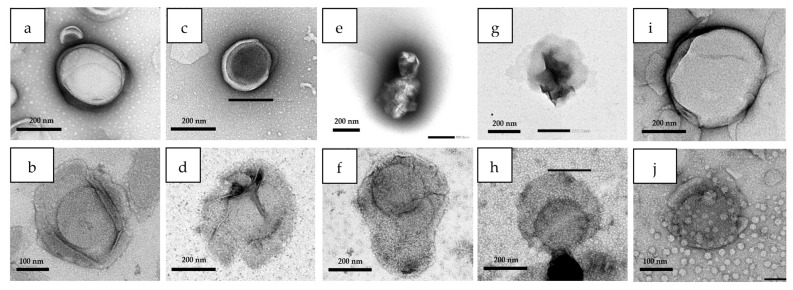
TEM images (**a**) SGC-Lip in SGF, (**b**) ES100-SGC-Lip in SGF, (**c**) SGC-Lip in FaSSIF, (**d**) ES100-SGC-Lip in FaSSIF, (**e**) SGC-Lip in FeSSIF, (**f**) ES100-SGC-Lip in FeSSIF, (**g**) Lip in FeSSIF, (**h**) ES100 -Lip in FeSSIF, (**i**) SGC-Lip in PBS pH 7.4, (**j**) ES100-SGC-Lip in PBS pH 7.4 after 2-h incubation at 37 °C.

The FTIR spectra and TEM images of Lip, SGC-Lip, ES100-Lip, and ES100-SGC-Lip in different release media are shown in Figure 7 and Figure 8. Figure S6 shows photo images of different liposomal formulations in different release media. These results explained the disintegration process of liposome formulation partially as discussed below.

### 3.7. Stability Study

**Figure 9:**
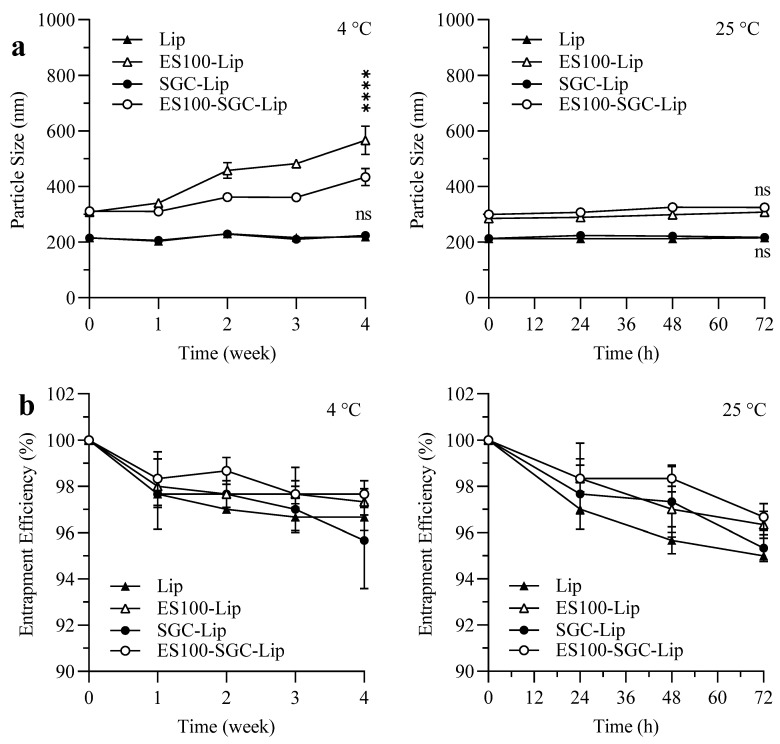
The effect of short- and long-term storage on the (**a**) particle size and (**b**) EE of different liposomal formulations at 25 °C and 4 °C for 72 h and 4 weeks, respectively. **Notes:** n = 3. (ns *p* ≥ 0.05, **** *p* < 0.0001) when SGC-Lip is compared to Lip and ES100-SGC-Lip is compared to ES100-Lip.

The results in Figure 9 showed that Lip and SGC-Lip vesicles incubated at room temperature and at 4 °C for 3 days and 4 weeks, respectively, did not show a significant change in particle size while showing a slight decrease in EE (>95%). Coated formulations (ES100-Lip and ES100-SGC-Lip) showed similar results at 4 °C, but they displayed a gradual increase in particle size after 4 weeks of storage. After 4 weeks, ES100-SGC-Lip vesicles increased in size from 310 nm to around 430 nm, while those of ES100-Lip increased to around 570 nm.

## 4. Discussion

### 4.1. Effect of SA on Liposome Particle Size, PDI, EE, and Zeta Potential

Different molar ratios of SA were used in the formulations of liposomes to increase the cationic surface charge of the vesicles to facilitate coating with the anionic polymer ES100. The goal was to achieve the highest positive charge with the minimum SA concentration without compromising other liposome properties (particle size, EE, safety, etc.).

Liposomes without SA showed an extensive degree of aggregation, probably due to the low surface charge that causes electrostatic attraction among liposomes, lowering liposomal dispersibility and stability [37]. The inclusion of SA into liposomes increased the size distribution homogeneity and prevented the aggregation of liposomes (Figure 2a,b). Liposomes with a 0.25 molar ratio of SA showed less aggregation and significantly lower particle sizes and PDI due to the increased electrostatic repulsion between the charged vesicles. Increasing the molar ratio of SA above 0.5 had no significant effect on the particle size and PDI, other than suppressing aggregation, which is compatible with the findings of other research [32].

As the SA molar ratio increased from 0 to 1, an increase in BUD EE was observed (Figure 2c), which could be a result of the formation of more dispersed populations of liposomes as the SA molar ratio increased, increasing the opportunity for entrapping the drug [38]. There is also evidence that the inclusion of SA (molar ratio of 0.5 or 5 mol%) can change the shape of DPPC:SA liposomes from multilamellar vesicles (MLVs) to unilamellar vesicles (LUVs), which increases EE significantly [39]. Further increases in the SA molar ratio decreased the EE of the drug, possibly due to the consequent decrease in the molar ratio of DPPC. Higher SA concentrations can disrupt the structure of the liposomes since it lacks the amphiphilic nature of phospholipids [40].

The increase in zeta potential shown in Figure 2d was expected due to the increase in the cationic surface charge as the SA molar ratio increased, up to a specific point at which the liposomal surface is saturated with SA (when the SA molar ratio is more than 3). An SA molar ratio of 1 is sufficient for the goal of the experiment; however, we expected that the inclusion of the bile salt might decrease the zeta potential slightly. Therefore, an SA molar ratio of 3 was chosen for the next step in liposome formulation (optimization of the bile salt concentration).

### 4.2. Effect of Bile Salt and CH Content on Liposome Particle Size, EE, and Zeta Potential

Among several bile salts (SGC, SDC, or STC) that could be used for the purpose of this research, SGC has the advantage of being an inhibitor of the main proteases in the GIT: pepsin, trypsin, and α-chymotrypsin. In addition, the safety profile of SGC is better [25].

The particle size of liposomes increased significantly as SGC concentration increased in all three formulations regardless of the method of preparation (sonication or extrusion) (Figure 3(a1,a2)). There have been conflicting findings about the effects of bile salts on particle size, with some studies finding that increasing the SDC concentration can reduce the particle size and PDI due to reduced surface tension of the vesicles [28], while other research has shown that increasing the SDC concentration can increase the liposome particle size by increasing the EE of the drug [24,41], or due to the bulkiness of the steroid-like structure of the bile salt [42].

The negative effect of SGC on drug EE of liposomes prepared by sonication (Figure 3(b1)) could be attributed to the competition between BUD and the structurally similar surfactant in the lipid bilayer, which might exclude the drug from the bilayer [43]. This effect is usually observed in more lipophilic surfactants; however, the presence of a positive charge on the liposome might preferably attract and incorporate the anionic surfactant SGC in the bilayer at the expense of the drug [44,45].

Liposomal formulations prepared by extrusion showed much lower EE than formulations prepared by sonication (Figure 3(b2)). This could be due to the loss of the drug inside the inner layers of non-extruded liposomes to the aqueous medium during the extrusion process, where the lipid bilayer is undergoing fragmentation, structural rearrangement, and in the case of DPPC, interdigitation [46,47].

The positive effect of SGC on the EE of BUD in liposomes prepared by extrusion can be explained by the surface-active properties of the bile salt, where they can disrupt the acyl chains of the phospholipids and increase the solubility of lipophilic drugs in the lipid bilayer [48]. However, increasing the SGC concentration further started to decrease the EE again, indicating that the capacity of solubilization by SGC was limited, and the additional SGC might begin to compete with the drug for the lipid bilayer [28,44]. There was a possibility that SGC might increase the solubility of BUD in the aqueous phase rather than the lipid bilayer, therefore an additional experiment was performed where the solubility of BUD was measured in different concentrations of SGC (0, 0.25, 0.5, and 1 mg/mL) in 5% dextrose. There was no significant difference in the solubility of BUD in the above-mentioned concentrations of SGC.

Similar to SGC, the effect of CH on EE was negative in liposomal samples prepared by sonication (Figure 3(b1)) and positive with samples prepared by extrusion (Figure 3(b2)). On the one hand, CH inclusion has been described as decreasing the permeability and increasing the encapsulation of hydrophilic drugs [49,50,51]. On the other hand, both CH and BUD have a similar lipophilic steroidal structure and can compete for the same lipophilic sites in the liposome membrane. CH is more lipophilic (log p ∼ 7.17) [52] than BUD (log p ∼ 3.21) [53] and therefore is favorably incorporated into the lipid bilayer [47,54].

The decrease in zeta potential in formulations prepared by sonication (Figure 3(c1)) when bile salts were incorporated was possibly due to the insertion of negatively charged glycocholate into the lipid bilayer [22].

A DPPC:CH:SA molar ratio of 7:3:3 and SGC concentration of 0.25 mg/mL were chosen for the next step, from which all formulations (SGC-Lip: uncoated liposomes with bile salt) were prepared exclusively by extrusion.

### 4.3. Effect of Drug/Lipid Weight Ratio on EE

Different drug/lipid weight ratios were tested to encapsulate as much drug as possible efficiently into the liposomes. The results showed that the higher the lipid ratio, the higher the EE, which is in agreement with other studies [54,55,56]. The increase in EE as the drug/lipid ratio decreased (low drug concentration) can be explained by the relative abundance of the lipid bilayer vacant sites that can accommodate the drug molecules, thus facilitating drug loading, leading to high EE. Conversely, when the drug/lipid ratio increases (high drug concentration), the lipophilic space of the lipid bilayer would be rapidly saturated, leaving the residual drug molecules in the aqueous phase, resulting in low EE [54]. A drug/lipid ratio of 1:40 was thus chosen for the next step.

### 4.4. Liposome Coating

**Figure 10:**
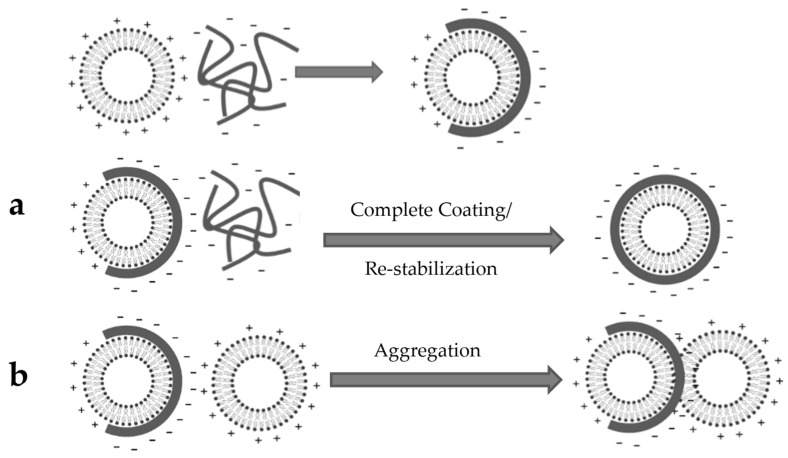
Schematic diagram of the interaction between the liposomes and ES100. The fate of interaction can either end with (**a**) complete coating, charge reversal, and restabilization, (**b**) or be interrupted by aggregation.

The interaction between a charged particle and a strong polyelectrolyte of the opposite charge has been explained by the “charge mosaic” model [57,58]. Depending on experimental conditions, the interaction between ES100 and the liposomes (irreversible coating) can lead to either particle restabilization or particle aggregation (Figure 10) [36]. Restabilization occurs when the interaction between the polymer and liposome proceeds until the charge of the polymer-coated particle is completely reversed. Conversely, aggregation can occur when a partly-coated liposome interacts with a non-coated liposome, which leads to particle agglomeration and an increase in measured particle size. These two reactions occur simultaneously in competition, which explains why it is difficult to avoid some aggregation [59].

Table 3 shows the parameters that were tested to optimize the coating process by increasing the rate of restabilization over the rate of aggregation. Based on previous studies [35,59], for the purpose of complete coating and restabilization, adding liposomes to the ES100 solution (and not the reverse order of addition) is preferred to make an excess of polymer available instantaneously for the liposomes.

At low liposomal lipid concentrations, fewer liposomes are presented to an excess of ES100, and therefore the rate of coating is increased while minimizing collisions between liposome particles. As such, less aggregation is expected, and a decrease in particle size measurement is observed (Figure 4a) [60]. For the same reason, decreasing the liposome/polymer ratio led to a slight but statistically significant decrease in particle size (Figure 4b). A high polymer concentration coupled with optimum coating conditions ensures complete liposome coating/restabilization with less opportunity for aggregation, and hence a lower particle size was detected (Figure 4c and Figure S2c). The apparent increase in the measured size when coating with low concentration ES100 solution could be explained by the agglomeration of two or more vesicles packed together, which cannot be distinguished by DLS [35]. Adding the liposomes faster to the polymer solution decreased particle size (Figure 4d) and PDI (Figure S2d) significantly, probably as a result of suppressing aggregation. With a slow rate of addition, the liposomes added first will be partially coated with negatively charged ES100 and can easily interact with cationic liposomes added later, resulting in aggregation. In contrast, adding liposomes faster will ensure that liposomal surface reversal occurs at the same time in all liposomes, maintaining repulsion between dispersed nanoparticles [36]. Similarly, increasing the rate of mixing helps the liposomes to be surrounded with fresh polymer particles available for interaction, enhancing the rate of coating while preventing liposome aggregation and causing a decrease in particle size (Figure 4e) and PDI (Figure S2e) [61].

As expected, increasing the SA molar ratio can increase the positive charge of the liposome surface, which facilitates interactions with the anionic polymer resulting in faster coating, with less change for aggregation.

The zeta potential measurement shown in Figure S3 did not significantly change when different coating variables were tested, except for the ES100 concentration. However, the difference in zeta potential was not as large as expected, possibly due to the extensive aggregation of liposomes, which led to a broad size distribution not suitable for the DLS technique. There was a slight decrease in the EE of BUD after coating, which could be a result of the displacement of some of the adsorbed BUD from liposome surfaces by ES100 during the coating [36].

In summary, after coating SGC-Lip with ES100 (ES100-SGC-Lip: coated liposomes with bile salt), particle size and PDI increased from 213 nm and 0.087 to 275 nm and 0.128, respectively. The zeta potential decreased from +53 mV to −38 mV. The EE of BUD was 95% and 91% before and after the coating, respectively.

FTIR spectra of ES100, SGC-Lip, and ES100-SG-Lip were obtained to support the formation of the ES100 coat around the liposomes. Figure S4 shows the characteristic peaks of ES100 and phospholipids. ES100 characteristic peaks include C=O ester stretching at 1730 cm^−1^, –CH_3_ bending at 1450.7 cm^−1^, and C–O ester stretching at 1149 cm^−1^. The liposome spectrum is characterized by phospholipoid signals including C=O ester stretching at 1740 cm^−1^ and P=O stretching at 1090 cm^−1^ [62,63,64]. ES100-SGC-Lip show the C–O and P=O peaks at both the characteristic wavenumbers (1149 cm^−1^ and 1090 cm^−1^) of ES100 and liposomes, respectively, confirming the formation of the ES100 coating layer around liposomes.

TEM images showed the spherical shape of the liposomes (Figure S5). The diameter of the liposomal formulations (Lip, SGC-Lip, and ES100-SGC-Lip) observed by TEM images matched with the size obtained from DLS. Both Lip and SGC-Lip formulation showed non-aggregating spherical vesicles with a smooth surface. However, the ES100-SGC-Lip formulation micrograph showed the presence of several clusters ranged in size from a few liposomes to large aggregates, but individual vesicles were also observed. The ES100 coating layer surrounding the surface of the liposome was visible and the coating thickness was in good agreement with the particle size measurement of coated liposomes (25–50% increase in size).

Overall, there was considerable evidence to confirm that the liposomal formulations were successfully coated with ES100 (Figure 4, Figures S4 and S5).

### 4.5. Drug Release Study

To examine the protection capability of SGC and ES100 in the GIT, the release of different liposomal formulations (Lip, SGC-Lip, ES100-Lip, and ES100-SGC-Lip) was tested in simulated GI fluids, as shown in Table 2. Lip and ES100-Lip formulations represent uncoated liposomes and coated liposomes, respectively, both without bile salt.

As shown in Figure 5, initially, a burst release of BUD was observed in uncoated formulations, most likely as a result of the release of BUD adsorbed on the liposome surface as well as the free BUD molecules. After that, a slower and sustained release occurred because of the BUD entrapped in the liposomes. A previous study showed that the release kinetics of a model lipophilic drug (curcumin) from DPPC/CH liposomes were found to be the best fit to the Korsmeyer–Peppas model, with Fickian diffusion as the predominant drug release mechanism [65]. Most of the drug (>90%) was released from SGC-Lip after a 2-h incubation in SGF and a 4-h incubation in FaSSIF, demonstrating that the use of uncoated liposomes would not be a viable option as a delivery vehicle to the colon. In contrast, ES100-SGC-Lip displayed slow and decreased drug release in SGF and FaSSIF compared to SGC-Lip. The release of the drug from the ES100-SGC-Lip formulation was pH dependent, with more drug being released as the pH of the medium was increased. In SGF and FaSSIF, less than half of the drug was released within 2 and 4 h, respectively, demonstrating the potential for ES100-SGC-Lip to prevent premature drug release in the environment of the stomach and upper intestine. In PBS pH 7.4, ES100-SGC-Lip allowed the release of most of the drug due to the rapid dissolution of the ES100 coating layer within 60 min at pH 7.4 and 37 °C [66]. The onset of degradation of Eudragit S100 films has been shown to occur within minutes at pH >7, similar to that observed in the current study [67].

Since bile salts in the GI tract can accelerate the degradation of liposomes leading to premature drug release, the drug release profiles were tested in the presence of bile salts by using the FeSSIF (Figure 5c). STC was chosen as a model bile salt because cholic acid is one of the more predominant bile salts in human bile [68]. Two additional formulations (Lip and ES100-Lip) were tested as a control in FeSSIF to examine the effect of bile salt inclusion into the liposomal formulations (SGC-Lip and ES100-SGC-Lip). The ES100 coating allowed the coated formulations (ES100-Lip and ES100-SGC-Lip) to be resistant to attack by bile salts compared to uncoated formulations (Lip and SGC-Lip), which underwent total destruction after 1 h of incubation in FeSSIF. The difference in drug release between formulations that contain bile salt and others that do not (SGC-Lip vs. Lip and ES100-SGC-Lip vs. ES100-Lip) is probably attributed to the protective effect of the bile salt that was incorporated into the SGC-Lip and ES100-SGC-Lip formulations. In previous research, the integrity and stability of bile salt-containing liposomes were examined in SGF, FaSSIF, FeSSIF, and ex vivo GI enzyme fluid. The results also revealed that SGC-liposomes displayed better integrity and stability than conventional liposomes [22]. Another study reported that ceftriaxone leakage from conventional liposomes was significantly higher than that from bile salt-containing liposomes in FeSSIF and FaSSIF after 4 h of incubation [24]. Further work involving in vivo studies in animals are being explored to evaluate the formulation efficacy in a more complex environment.

The release of different liposomal formulations was tested in distinct simulated GI fluids in the present study. However, the drug release was studied independently in each medium, which does not fully simulate the physiological conditions in the GIT. Ramalho et al. conducted a sequential drug release study from PLGA nanoparticles in simulated media, where the same formulation batch is transferred from one medium to another, mimicking the fate of the formulation in the GIT [69]. This experimental model may further clarify the drug release from ES-100-SGC-Lip in simulated physiological media in a more representative manner.

### 4.6. Liposomal Integrity

Liposomal integrity was tested in terms of changes in particle size, morphology, and coating stability in each of the release media (SGF, FaSSIF, FeSSIF, PBS pH 7.4) (Figure 6, Figure 7 and Figure 8). Particle size measurements after incubation in different release media are illustrated in Figure 6. The overall particle size of both SGC-Lip and ES100-SGC-Lip were retained with minimal variation in SGF and FaSSIF (Figure 6a,b). Both SGC-Lip and ES100-SGC-Lip vesicle particle sizes increased in PBS pH 7.4 within 60 min (Figure 6d). Regarding the uncoated liposomes, DPPC liposome size generally increased with increasing pH and temperature of the medium due to the deprotonation of the phosphate group of the lipid at high pH, causing electrostatic repulsion between the hydrophilic head and an increase in particle size [65]. For coated liposomes, the same effect of increasing the particle size as a result of increasing the pH is expected; however, it is also expected that the dissolution of the coating layer at pH 7.4 would reduce the particle size as seen in the work of Kim, H. Y. et al. (2020) [17]. A possible explanation for the increase in the particle size of coated liposomes is the precipitation of the dissolved ES100 layer due to the low ionic strength of the PBS buffer (~10 mM) compared to the high ionic strength of the phosphate buffer pH 8 (100 mM) in which the polymer was dissolved during preparation. This explanation is supported by the photo images in Figure S6 that show the precipitated polymer in PBS pH 7.4 and TEM in Figure 8 that shows the polymer nanoprecipitate. In FeSSIF, four formulations were tested for particle size changes (Lip, SGC-Lip, ES100-Lip, ES100-SGC-Lip) as shown in Figure 6c. The uncoated formulations’ (Lip and SGC-Lip) particle size measurements did not meet the DLS criteria because of the total destruction of the liposomes, while the coated formulations (ES100-SGC-Lip and ES100-Lip) retained their particle size without change due to the protective effect of the ES100 coating layer.

FTIR spectra showed structural changes in coated liposomes (Figure 7). The intensity of the characteristic ES100 peak of C–O ester stretching at 1149 cm^−1^ was changed in relation to other peaks in each release medium. The intensity of the peak decreased in the release media with the order of SGF > FeSSIF > FaSSIF > PBS pH 7.4. The peak intensity of C–O ester is partially correlated with the amount (per unit volume) of the functional group in ES100 [70]. The decrease in the peak intensity as the pH of the release media is increasing indicates the removal of the ES100 coating layer during the incubation. These results support the pH-dependent dissolution of the outer coating layer of ES100-SGC-Lip. As mentioned before, there were still some ES100 nanoprecipitates that were suspended in the formulation after dissolution of the coating layer, which manifested as small peaks in the FTIR spectrum of PBS pH 7.4.

The structural and morphological changes of different liposomal formulations were examined by TEM after a 2-h incubation in each release medium. TEM images in Figure 8 showed that SGC-Lip and ES100-SGC-Lip did not undergo an obvious shape change after 2-h incubation at the SGF and FaSSIF, which coincided with the particle size measurement. In PBS pH 7.4, SGC-Lip showed an increase in the liposome vesicle, with no sign of aggregation, which is also in agreement with the particle size measurement. In the case of ES100-SGC-Lip, TEM images showed the complete dissolution of the ES100 coating layer into visible fragments in the nano range, which explain the presence of the ES100 characteristic peak after dissolution in FTIR analysis. TEM images of four formulations (Lip, SGC-Lip, ES100-Lip, and ES100-SGC-Lip) were also obtained in FeSSIF (Figure 8), where Lip and SGC-Lip appeared to have lost their vesicle structure and integrity, while ES100-Lip and ES100-SGC-Lip showed no difference in morphology or structure after the 2-h incubation.

### 4.7. Stability Study

Liposomes can be unstable for several reasons; liposome particle size can increase during storage due to increasing liposomal aggregation or fusion [36]. Additionally, drug EE can decrease as a result of the drug leaking out of liposomes during storage [71]. To ensure optimal liposomal function and therapeutic efficacy throughout the storage period, the stability of the liposomes was studied by monitoring their size and EE at 25 °C and 4 °C (Figure 9).

The results in Figure 9 show that Lip and SGC-Lip vesicles at room temperature and at 4 °C were essentially stable for 3 days and 4 weeks, respectively. Coated formulations (ES100-Lip and ES100-SGC-Lip) showed similar results at 4 °C; however, their particle size gradually increased after 4 weeks of storage. However, there is a significant difference between ES100-SGC-Lip and ES100-Lip vesicle particle size after 4 weeks, where ES100-Lip vesicles nearly double in size compared to ES100-SGC-Lip vesicles, which displayed a 40% increase in size. The increase in particle size might be due to the slow aggregation of liposomes due to the interaction between the anionic-coated liposomes and the positive surfaces of liposomes with a defective coating. The relative stability of the ES100-SGC-Lip formulation compared to ES100-Lip might be due to the negative charge induced by SGC on the surface of the liposomes, which caused electrostatic repulsion that prevented the fusion and aggregation of vesicles during storage [27].

## 5. Conclusions

A liposomal formulation suitable for colonic targeting via oral administration could provide new opportunities for local and systemic drug delivery. Among the developed formulations, ES100-SGC-Lip showed a high EE and a narrow size distribution. Standardization of the lipid and polymer concentrations, the rate of liposome addition and mixing, and the liposomal surface charge were essential for efficient and uniform coating. ES100-SGC-Lip prevented premature drug release in SGF, FaSSIF, and FeSSIF while showing a pH-dependent dissolution of the coating layer in PBS pH 7.4, followed by the subsequent release of the drug within the average transit time of the colon. These results demonstrated in vitro that the formulation has potential as a colon-targeted delivery system.

## Figures and Tables

**Table 1:** Compositions of various liposomal formulations. The quantities mentioned below were used to prepare 1 mL of liposomal dispersions (20 mM).

DPPC:CH:SA Molar Ratio	DPPC (mg)	CH (mg)	SA (mg)	BUD (mg)
7:3:0	11.0	2.3	0.0	0.25
7:3:0.25	10.7	2.3	0.1	0.25
7:3:0.5	10.5	2.2	0.3	0.25
7:3:1	10.0	2.1	0.5	0.25
7:3:2	9.2	1.9	0.9	0.25
7:3:3	8.5	1.8	1.2	0.25
7:3:4	7.9	1.7	1.5	0.25
8:2:3	9.7	1.2	1.2	0.25
6:4:3	7.2	2.4	1.2	0.25

**Table 2:** Compositions of simulated physiological fluids used as release media.

Ingredient	SGF	FaSSIF	FeSSIF	PBS
NaCl	2 g	6.19 g	11.87 g	8 g
KCl				0.2 g
NaOH pellet		0.35 g	4.04 g	
Glacial acetic acid			144 mM	
STC			10 mM	
NaH_2_PO_4_		3.44 g		
NaH_2_PO_4_				1.15 g
KH_2_PO_4_				0.2 g
HCl conc.	7 mL			
H_2_O qs.	1000 mL	1000 mL	1000 mL	1000 mL
pH	1.2	6.5	5	7.4

**Table 3:** Tested parameters that potentially affect the coating process. To isolate the effect of each coating variable under test (from row 1 to 6), the other parameters in each row were fixed.

No.	Coating Variables	Lipid Concentration (mM)	Liposome/Polymer Volume Ratio	Polymer Concentration (mg/mL)	Rate of Lipid Addition (µL/s)	Mixing Speed (rpm)	SA Molar Ratio
**1.**	**Lipid Concentration** **(mM)**	2, 4, 5, 6, 7, 10	1:4	2	250	1000	3
**2.**	**Liposome/Polymer** **Volume Ratio**	5	1:1, 1:2, 1:3, 1:4	2	250	1000	3
**3.**	**Polymer Concentration (mg/mL)**	5	1:1	0.25, 0.5, 1, 2, 2.5	250	1000	3
**4.**	**Rate of Lipid Addition** **(µL/s)**	5	1:1	2.5	5, 10, 50, 250	1000	3
**5.**	**Mixing Speed** **(rpm)**	5	1:1	2.5	250	250, 500, 1000	3
**6.**	**SA Molar** **Ratio**	5	1:1	2.5	250	1000	1, 2, 3

## Data Availability

Not applicable.

## Supplementary Materials

The following supporting information can be downloaded at: https://www.mdpi.com/article/10.3390/polym14132693/s1, Figure S1: The effect of drug-to-lipid weight ratio on the EE of liposomes prepared by extrusion. DPPC:CH:SA molar ratio was 7:3:3 and SGC concentration was 0.25 mg/mL. Notes: The data represent the mean ± standard deviation (n = 3). (**** *p* < 0.0001) compared to adjacent formulation(s); Figure S2: The effect of various coating variables: (a) lipid concentration, (b) lipid–polymer volume ratio, (c) ES100 concentration, (d) rate of lipid addition, (e) mixing speed, (f) SA molar ratio on the PDI of liposomes. Notes: The data represent the mean ± standard deviation (n = 3). (ns *p* ≥ 0.05, * *p* < 0.05, ** *p* < 0.005, *** *p* < 0.001, **** *p* < 0.0001) compared to adjacent formulation(s); Figure S3: The effect of various coating variables: (a) lipid concentration, (b) lipid–polymer volume ratio, (c) ES100 concentration, (d) rate of lipid addition, (e) mixing speed, (f) SA molar ratio on the zeta potential of liposomes. Notes: The data represent the mean ± standard deviation (n = 3). (ns *p* ≥ 0.05, * *p* < 0.05, *** *p* < 0.001) compared to adjacent formulation(s); Figure S4: Fourier transform infrared spectra of Eudragit S100 polymer and the liposomal formulation before and after coating with the polymer; Figure S5: Transmission electron microscopy images of (a) Lip, (b) SGC-Lip, (c) ES100-SGC-Lip. From top left to bottom right, bars represent 100 nm, 200 nm, 100 nm, 200 nm, and 1µm; Figure S6: Photo images of various liposomal formulations after 24-h incubation at 37 °C in (a) SGF pH 1.2, (b) FaSSIF pH 6.5, (c) FeSSIF pH 6.5, (d) PBS pH 7.4.
