# Supplementary material for: Preparation, Characterization and In Vitro Evaluation of Eudragit S100-Coated Bile Salt-Containing Liposomes for Oral Colonic Delivery of Budesonide
Source: Polymers (Basel). 2022 Jun 30;14(13):2693. doi: 10.3390/polym14132693 (PMC9268925; doi:10.3390/polym14132693)
Supplement: Supplementary file 1 [file polymers-14-02693-s001.zip › polymers-1747456-supplementary.pdf]

polymers

MDPI

Preparation, Characterization, and In Vitro Evaluation of Eudragit S100-Coated Bile Salt-Containing Liposomes for Oral Colonic Delivery of Budesonide

Hamid Alghurabi [1,2], Tatsuaki Tagami [1], Koki Ogawa [1] and Tetsuya Ozeki [1,*]

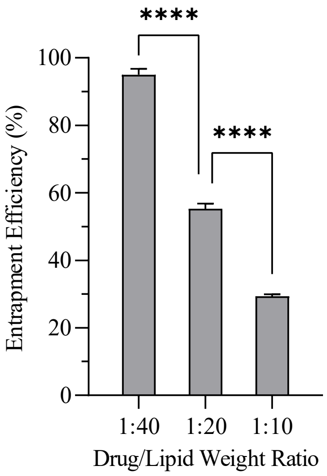

Figure S1. The effect of drug-to-lipid weight ratio on the EE of liposomes prepared by extrusion. DPPC:CH:SA molar ratio was 7:3:3 and SGC concentration was 0.25 mg/mL. **Notes**: The data represents the mean ± standard deviation (n = 3). (**** $p < 0.0001$) compared to adjacent formulation(s).

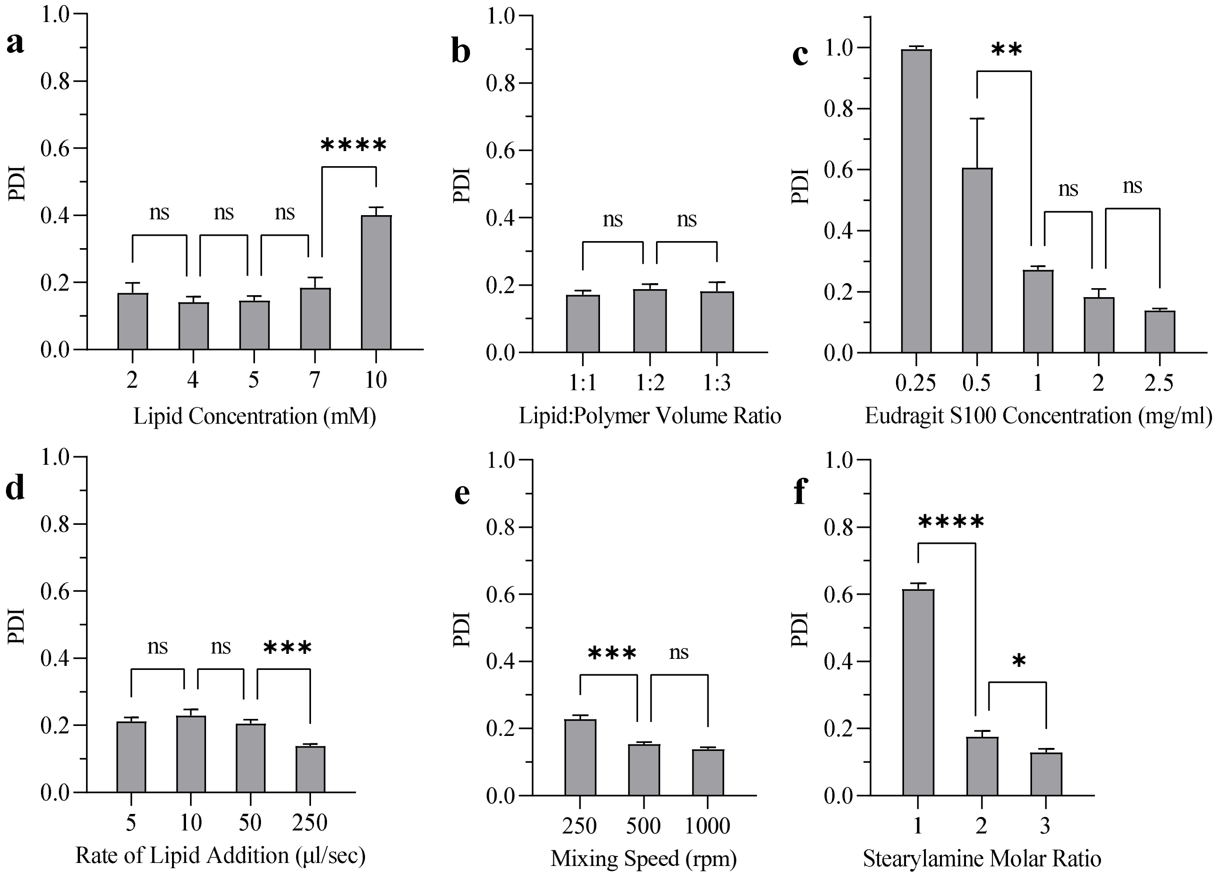

Figure S2. The effect of various coating variables: (**a**) lipid concentration (**b**) lipid:polymer volume ratio (**c**) ES100 concentration (**d**) rate of lipid addition (**e**) mixing speed (**f**) SA molar ratio on the PDI of liposomes. **Notes**: The data represents the mean ± standard deviation (n = 3). (ns $p \geq 0.05$, * < 0.05, ** $p < 0.005$, *** $p < 0.001$, **** $p < 0.0001$) compared to adjacent formulation(s).

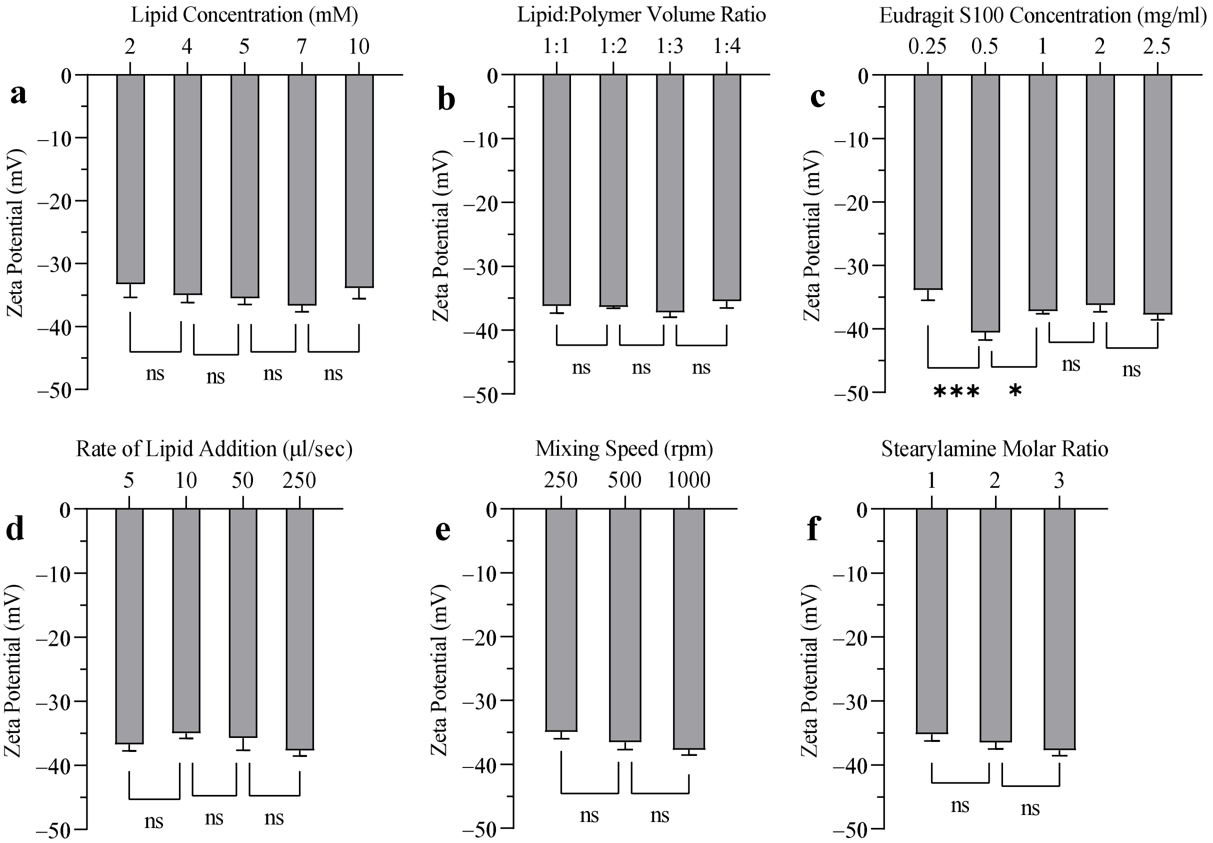

Figure S3. The effect of various coating variables: (**a**) lipid concentration (**b**) lipid:polymer volume ratio (**c**) ES100 concentration (**d**) rate of lipid addition (**e**) mixing speed (**f**) SA molar ratio on the zeta potential of liposomes. **Notes**: The data represents the mean ± standard deviation (n = 3). (ns $p \geq 0.05$, * $p < 0.05$, *** $p < 0.001$) compared to adjacent formulation(s).

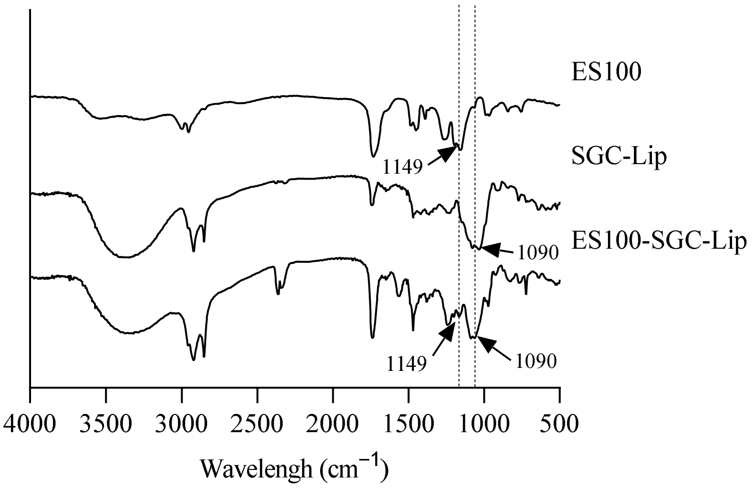

Figure S4. Fourier-transform infrared spectra of Eudragit S100 polymer and the liposomal formulation before and after coating with the polymer.

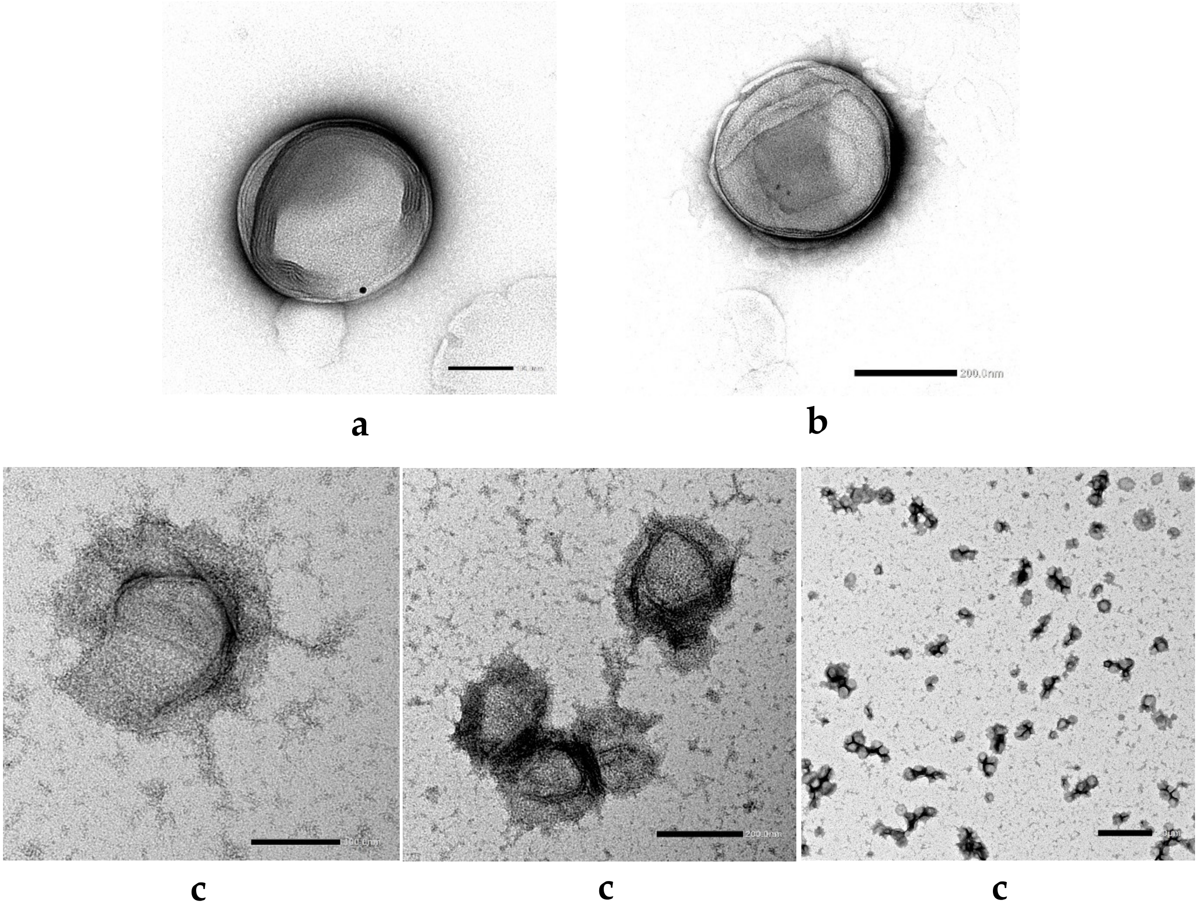

Figure S5. Transmission electron microscopy images of (**a**) Lip (**b**) SGC-Lip (**c**) ES100-SGC-Lip. From top left to bottom right, bars represent 100 nm, 200 nm, 100 nm, 200 nm, and 1 μm.

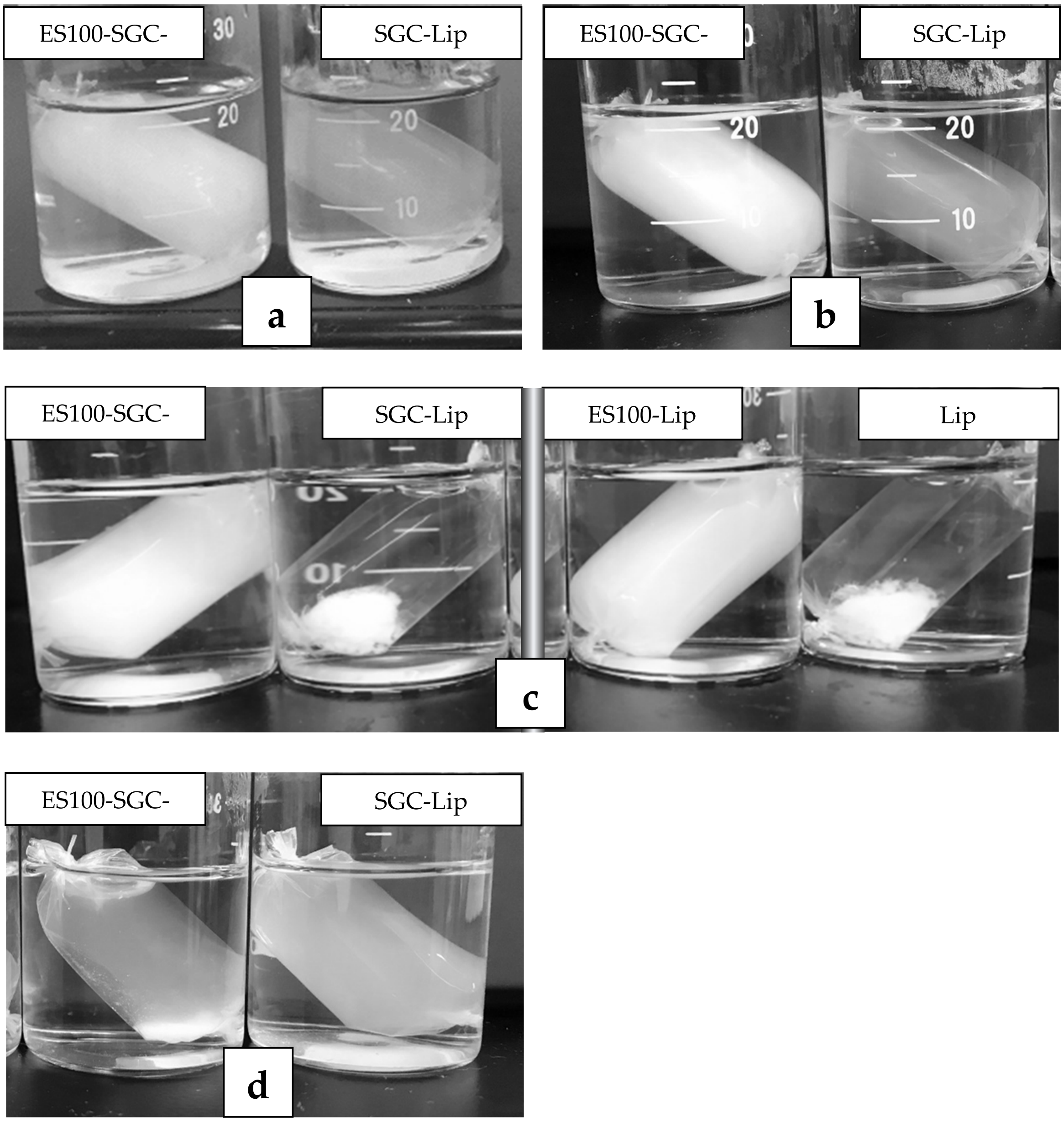

Figure S6. Photo images of various liposomal formulations after 24 h incubation at 37 °C in (**a**) SGF pH 1.2 (**b**) FaSSIF pH 6.5 (**c**) FeSSIF pH 6.5 (**d**) PBS pH 7.4.
